# Evaluating the Neuroprotective Effects of Levetiracetam on Experimental Sciatic Nerve Injury

**DOI:** 10.3390/jcm14186374

**Published:** 2025-09-10

**Authors:** Duygu Demiriz Gulmez, Gulay Hacioglu, Esma Cinar, Arif Keskin, Ozgun Cuvas Apan, Alparslan Apan

**Affiliations:** 1 Department of Anesthesiology and Reanimation, School of Medicine, Giresun University, Giresun 28200, Turkey; 2 Department of Physiology, School of Medicine, Giresun University, Giresun 28200, Turkey; 3 Department of Pathology, School of Medicine, Giresun University, Giresun 28200, Turkey; 4 Department of Anatomy, School of Medicine, Giresun University, Giresun 28200, Turkey

**Keywords:** peripheral nerve regeneration, levetiracetam, sciatic nerve, injury

## Abstract

**Background/Objectives:** Despite surgical interventions, patients experience incomplete nerve function recovery following peripheral nerve (PN) injuries. Levetiracetam (LEV), a third-generation antiepileptic agent and neuromodulator, has shown neuroprotective effects in conditions such as traumatic brain injury and diabetic neuropathy. However, its efficacy in PN injuries remains unexplored. **Methods:** We conducted an experimental study using 48 rats divided into six groups. After inducing sciatic nerve compression, LEV was administered intraperitoneally at 50 mg/kg/day for 7 days in the acute group and for 28 days in the chronic group. The sciatic functional index (SFI) was assessed on the 7th day in the acute group and on the 7th, 14th, 21st, and 28th days in the chronic group. Histological and tissue assessments were performed post-sacrifice (7th day for acute; 28th day for chronic). **Results:** Significant differences were observed among the groups in all parameters except capillary structure and inflammatory cell density. LEV-treated groups demonstrated higher GAP-43 and S-100 reactivity, improved SFI scores, and greater neuronal regeneration compared to non-drug groups (*p* < 0.05). LEV significantly enhances neuronal regeneration, protein expression (GAP-43 and S-100), and SFI outcomes, particularly in the chronic phase. **Conclusions:** This experimental animal study is the first to demonstrate LEV’s therapeutic potential in PN injury, providing a basis for further exploration.

## 1. Introduction

Peripheral nerves (PNs) are particularly susceptible to trauma owing to their anatomical positioning. While nerve injuries are often not life-threatening, they impose significant social and economic burdens by leading to temporary or permanent loss of function. Among the various types of nerve injuries, cuts, stretches, and compressions are the most prevalent, causing damage to peripheral axons, a condition known as Wallerian-like degeneration [1]. Activation of the immune system exacerbates Wallerian degeneration, hindering the regeneration of PNs [2,3].

Considering that inflammatory factors contribute to the acceleration of neurodegeneration, it is thought that anti-inflammatory substances can be used to prevent this effect, and there are various studies conducted in this direction [4,5,6]. However, there is no medical treatment that accelerates axonal regeneration and helps restore normal sensory and motor activities of the nerve. Therefore, new therapeutic strategies for PN repair are critical, and immunomodulators that increase repair aids while also suppressing harmful reactions may be promising.

Levetiracetam (LEV) [(S)-alpha ethyl-2-oxo-1-pyrrolidine acetamide] is a The Food and Drug Administration (FDA)-approved third-generation antiepileptic. LEV, a neuromodulator, changes neurotransmitter release by acting through binding to synaptic vesicle glycoprotein (SV2A) [7]. The neuroprotective properties of LEV, which has an anti-inflammatory effect and shows this effect by reducing the release of inflammatory cytokines TNF-alpha, IL-1 beta and IL-6, have also been demonstrated by various studies [6,8,9,10].

We aimed to evaluate the effect of LEV on trauma-related damage to PNs using the sciatic functional index (SFI) test, which has been proven to be a quantitative, reliable and economical method to evaluate the functional changes observed in the sciatic nerve, and the dry weight ratio of the gastrocnemius muscle.

## 2. Materials and Methods

This study adhered to the ARRIVE (Animal Research: Reporting of In Vivo Experiments) guidelines, and all protocols in this study were approved by the Committee on the Ethics of Animal Experiments of Giresun University (Permit number: 2019/17), in compliance with the Guide for the Care and Use of Laboratory Animals published by the US National Institutes of Health.

In our study, 48 adult male Wistar-Albino rats weighing between 250 and 350 g were used. The rats used in this study were obtained from “Saki Yenilli Laboratory for Experimental Animal Production and Applications”, a certified and accredited animal research facility. Rats were maintained under specific pathogen-free laboratory conditions on a 12 h light/dark cycle with free access to food and water. Rats were randomly selected among themselves. In order to evaluate the effect in the early (Groups Acute) and late period (Groups Chronic), two main groups were created, and each main group was divided into 3 subgroups: control, non-drug and drug groups. Each group included 8 rats.

### 2.1. Model

One of the most used models to evaluate the motor and sensory function of PNs is the sciatic nerve injury (SNI) model [11,12]. The most important reasons for choosing the sciatic nerve are that it is easily accessible, its surgery is relatively easy due to its large size, and it can be compared with previous studies. Crushing is applied to disrupt the continuity of all axons without disrupting the ligament scaffold of the nerve and thus losing the continuity of the nerve trunk. Therefore, nerve segments proximal and distal to the lesion site remain connected, allowing severed axons to regrow along an optimal regeneration pathway and reach their original innervation targets. PN damage causes the target muscle to atrophy as a result of denervation. If the muscle is reinnervated, atrophy stops, and muscle function begins a recovery process [13]. It can be considered that the closer the ratio of the healthy side to the damaged side is 1, the less muscle atrophy occurs. This experimental model is particularly suitable for investigating time course changes related to regeneration [12]. For this reason, we chose the SNI model in our study, using the SFI and the weight ratio of the gastrocnemius muscle innervated by the posterior branch of the SN for functional evaluation and aimed to observe the early and late effects of our agent.

### 2.2. Surgical Technique

**Figure 1:**
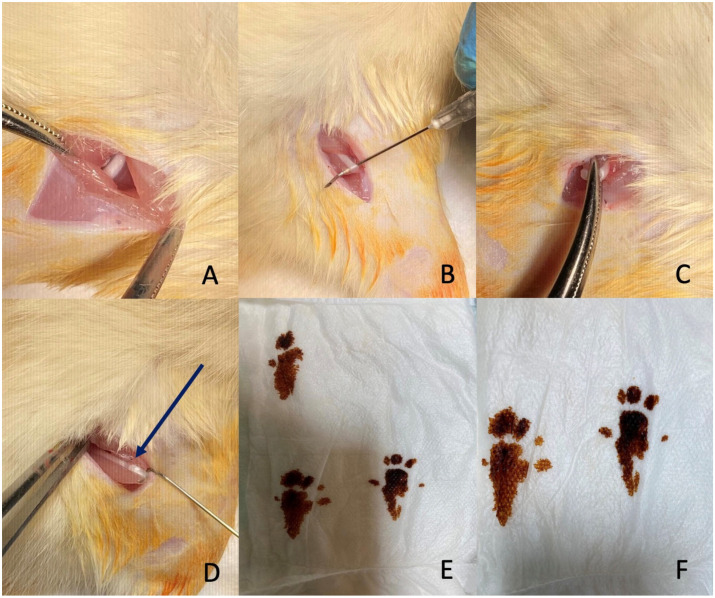
Surgical procedure and evaluation of sciatic nerve injury. (**A**,**B**) Exposure of the sciatic nerve through an incision and dissection. (**C**) Application of a clamp to induce compression injury to the nerve. (**D**) The crushed sciatic nerve showing the translucent band formation (arrow) indicating successful compression. (**E**,**F**) Footprints of the rats used to assess sciatic nerve function.

The rats were taken to the operating room and anesthetized by intraperitoneal administration of 80 mg/kg ketamine (Ketalar^®^, Pfizer Pharma GMBH, Karlsruhe, Germany) and 10 mg/kg xylazine hydrochloride (Alfazyne^®^, 2%, Alfasan International, 3440 AB, Woerden, The Netherlands). Anesthetic agents were repeated at half doses when necessary. After anesthesia was applied and stabilization was achieved, asepsis and antisepsis were maintained using a betadine solution. The right sciatic nerve was exposed in all rats in each group after an incision of the skin and gluteal muscle. In six experimental groups, the right sciatic nerve was compressed for 60 s using a 2 mm wide clamp, and complete crush was confirmed by the presence of a translucent band along the nerve (Figure 1). The incision was then closed in layers (muscle and skin) with absorbable sutures.

Following surgery, rats were treated as follows:

In the early (Acute groups) phase, no medication was administered to the control and non-drug groups. Intraperitoneal LEV was administered to the drug group at a dose of 50 mg/kg every day for 7 days. Eight rats from each group were randomly selected for SFI evaluation on days 3 and 7. On the 7th day, the rats were sacrificed and a 1 cm segment of the right sciatic nerve was collected from each rat for immunohistochemical examination.

In the late (Chronic groups) phase, no medication was administered to the control and non-drug groups. Intraperitoneal LEV was administered to the drug group at a dose of 50 mg/kg every day for 28 days. Eight rats from each group were randomly selected for SFI evaluation on the 7th, 14th, 21st, and 28th days. On the 28th day, the rats were sacrificed and a 1 cm segment of the right sciatic nerve was collected from each rat for immunohistochemical examination.

At the end of the experiment, euthanasia was performed in accordance with institutional and ethical guidelines for the humane treatment of laboratory animals. Animals were first anesthetized with an intraperitoneal injection of ketamine (80 mg/kg) and xylazine (10 mg/kg) to ensure deep anesthesia and the absence of pain or distress. Once a surgical plane of anesthesia was confirmed, euthanasia was completed by cervical dislocation.

### 2.3. Sciatic Nerve Function

Since walking is a complex process that requires motor unit denervation, the SFI, designed by Medinaceli et al., which evaluates the hind leg performance by examining the footprint, is a frequently used non-invasive method to determine the functional status in sciatic nerve damage [14]. Evaluation of SFI was performed on the 7th, 14th, 21st and 28th postoperative days. Rats were held by the chest and their hind legs were pressed against a stamp pad soaked in water-soluble blue ink. Rats were walked through a passage limited to a dark shelter at the end of the corridor. The walkway was lined with paper on the ground to catch possum footprints. The following measurements were taken from the footprints: (1) distance from heel to third toe, imprint length (PL), (2) distance from first to fifth toe to toe (TS), and (3) distance from second to fourth toe, intermediate toe (ITS). All three measurements were made from the experimental (E, sciatic nerve crush) and contralateral normal (N) limbs. The three factors that make up the SFI were calculated as follows: (1) print length factor (PLF) = (EPL − NPL)/NPL; (2) foot spread factor (TSF) = (EST − NST)/NST; and (3) middle finger spread factor (ITF) = (EIT − NIT)/NIT. Using these data, the SFI, which represents the differences between the injured and intact contralateral paw, was calculated with the following formula derived by Bain et al. [15]. An SFI equal to −100 indicates significant degradation, while an SFI oscillating around 0 was taken into account to reflect normal function:

SFI = −[38.3 × (EPL − NPL)/NPL] + [109.5 × (ETS − NTS)/NTS] + [13.3 × (EIT − NIT)/NIT] − 8.8

### 2.4. Gastrocnemius Muscle Weight Ratio

To evaluate sciatic nerve recovery, gastrocnemius muscles were dissected 7 days after surgery for acute groups and 28 days after surgery for chronic groups. The muscles were carefully collected from both the healthy and injured sides and weighed while wet. All measurements were made by two blind observers. The values were expressed as the ratio of the wet weight of the gastrocnemius muscle on the operated side to the wet weight of the muscle on the normal side.

### 2.5. Histopathological Examination

Each of the sciatic nerve samples was placed in a separate container and fixed in a 10% buffered formaldehyde solution. The samples were then placed in individual cassettes, and after tissue processing, they were embedded in paraffin, and 5-micron sections were obtained. The sections were stained using hematoxylin-eosin, S100 ([Polyclonal] FLEX RTU 6 mL), GAP-43 ([7B10] C.Liq. 0.2 mL (1:100)), and Toluidine blue (polychrome [150 mL]). Light microscopy (Olympus BX51, Olympus Corporation, Tokyo, Japan) was used for examination, and histological scoring was conducted based on the repair areas by a pathologist who was blinded to the group allocations, following the scale presented in Table 1.

**Figure 2:**
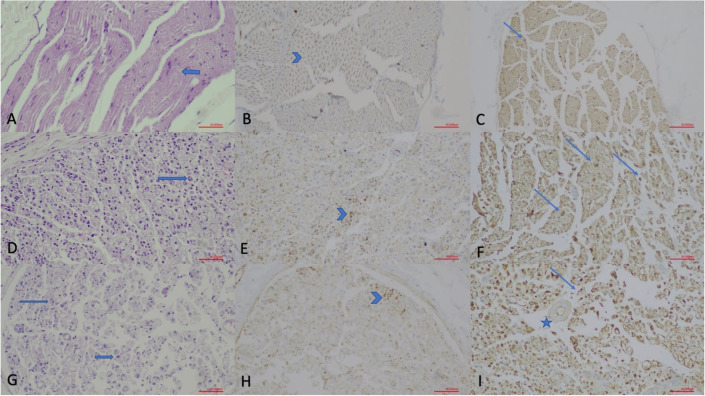
Histopathological evaluation of sciatic nerve sections in the acute phase (×200; scale bar = 50 µm). (**A**) Hematoxylin and Eosin (H.E.) staining of the Control Group, showing normal nerve morphology with intact muscle fibers and Schwann cell (arrow) nuclei. (**B**) GAP-43 immunostaining of the Control Group, demonstrating weak axonal staining (arrowhead). (**C**) S-100 staining of the Control Group, illustrating Schwann cell nuclei (thin arrows). (**D**) H.E. staining of the Non-drug Group, revealing disrupted nerve architecture and increased Schwann cell density (arrow). (**E**) GAP-43 immunostaining of the Non-drug Group, showing granular axonal staining (arrowhead) indicating early regeneration. (**F**) S-100 staining of the Non-drug Group, demonstrating increased Schwann cell activity (arrows). (**G**) H.E. staining of the Drug Group, showing reduced inflammation and improved structural preservation with increased Schwann cell nuclei (arrows). (**H**) GAP-43 immunostaining of the Drug Group, indicating strong granular axonal staining (arrowhead). (**I**) S-100 staining of the Drug Group, demonstrating marked Schwann cell proliferation (arrows) and a hypertrophic vascular wall (star).

**Figure 3:**
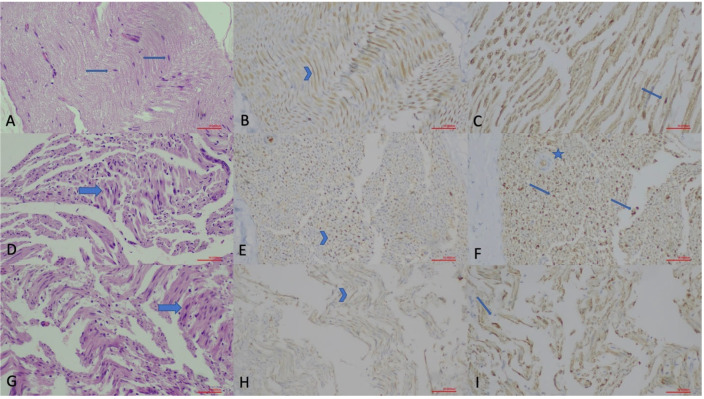
Histopathological evaluation of sciatic nerve sections in the chronic phase (×200; scale bar = 50 µm). (**A**) Hematoxylin and Eosin (H.E.) staining of the Control Group, showing normal nerve morphology with Schwann cell (arrow) nuclei. (**B**) GAP-43 immunostaining of the Control Group, demonstrating faint axonal staining (arrowhead). (**C**) S-100 staining of the Control Group, illustrating Schwann cell nuclei (thin arrow). (**D**) H.E. staining of the Non-drug Group, displaying disrupted nerve architecture with increased Schwann cell density (arrow). (**E**) GAP-43 immunostaining of the Non-drug Group, showing axonal staining (arrowhead) indicating regenerative activity. (**F**) S-100 staining of the Non-drug Group, demonstrating increased Schwann cell nuclei (arrows) and a hypertrophic vascular wall (star). (**G**) H.E. staining of the Drug Group, revealing preserved architecture and increased Schwann cell nuclei (arrow). (**H**) GAP-43 immunostaining of the Drug Group, showing mild axonal staining (arrowhead). (**I**) S-100 staining of the Drug Group, indicating increased Schwann cell nuclei (thin arrow).

Several histopathological techniques are available to assess the extent of PN regeneration. Key parameters include Schwann cell density, evidence of axonal regrowth, fiber and axon cross-sectional area, and myelin thickness [16]. Among these, demonstrating axonal regrowth is the most critical indicator of PN regeneration. Schwann cells play a vital role in PN regeneration but are challenging to identify using traditional histological methods. However, specific proteins such as S-100 and GAP-43 can accurately detect these cells [16]. Therefore, in our study, we utilized the analysis of these proteins to evaluate histopathological changes. Additionally, we assessed Schwann cell density, inflammatory cell density, and axonal continuity during the histopathological examination of the sciatic nerve (Figure 2 and Figure 3).

### 2.6. Statistical Analysis

The data were analyzed using SPSS version 22. The Shapiro–Wilk test was used to assess the normality of distribution. Data are presented as arithmetic mean (X), standard deviation, median, number, and percentage. One-way ANOVA, *t*-test, Kruskal–Wallis test, Friedman test, and Chi-square test were used for the analyses. Bonferroni and Tamhane tests were applied for post hoc analysis. A *p*-value of <0.05 was considered statistically significant.

## 3. Results

Table 2 summarizes the gastrocnemius muscle ratios and SFI measurements among the groups during the acute and chronic periods. In the acute phase, significant statistical differences were found between the gastrocnemius muscle ratios and the 7th-day SFI measurements (*p* < 0.001). Comparisons of acute phase muscle ratios and SFI measurements among sub-groups are detailed in Supplementary Table S1. Statistically significant differences in muscle ratios and SFI measurements were observed between the control-non-drug and control-drug groups (*p* < 0.001). However, no significant difference was detected between the non-drug and drug groups during the acute phase.

In the chronic phase, there were statistically significant differences in gastrocnemius muscle ratios and SFI values on the 7th, 14th, 21st, and 28th days (*p* < 0.001) (Table 2). Comparisons showed that muscle ratios and SFI values were significantly higher in the control group compared to the non-drug and drug groups (*p* < 0.05). Although the SFI values were lower in the drug group compared to the non-drug group on days 14, 21, and 28, this difference was statistically significant only on day 21 (*p* < 0.001) (Supplementary Table S2)

Comparisons of the SFI values in the chronic drug group over different time points revealed statistically significant differences (*p* < 0.001) (Supplementary Table S3). When SFI values and muscle ratios were compared in the acute and chronic drug groups, significant differences were observed, with SFI values being lower in the chronic drug group (Supplementary Table S4).

**Figure 4:**
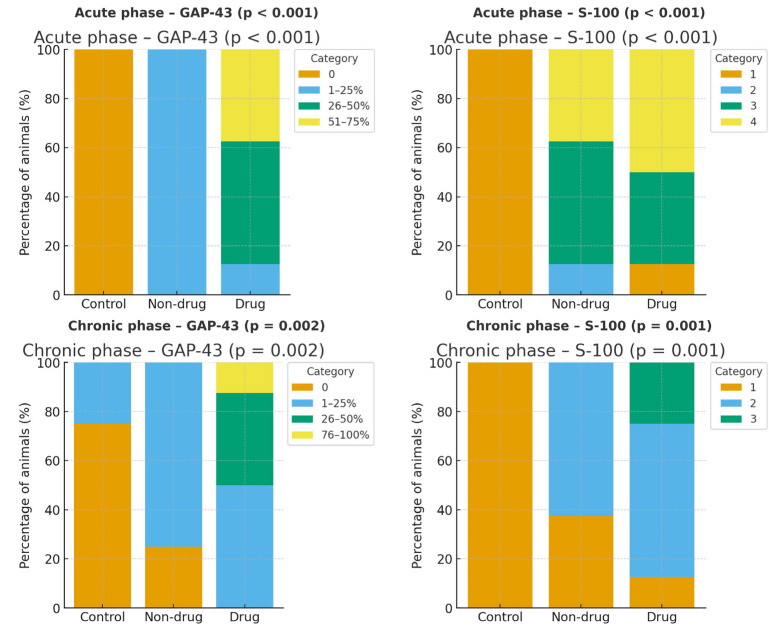
Distribution of GAP-43 and S-100 immunohistochemical staining across groups in the acute and chronic phases. Bars show the percentage of animals within each staining category (GAP-43: 0%, 1–25%, 26–50%, 51–75% for acute; 0%, 1–25%, 26–50%, 76–100% for chronic; S-100: ordinal scores 1–4 for acute and 1–3 for chronic). In the acute phase, GAP-43 staining was absent in controls and shifted toward higher categories with levetiracetam (LEV) (Drug) compared with Non-drug and Control; S-100 likewise shifted upward in Non-drug and Drug groups versus Control (overall χ^2^ *p* < 0.001 for both markers). In the chronic phase, the Drug group showed right-shifted GAP-43 distribution with some animals in the 26–50% and 76–100% categories, and higher S-100 scores relative to Control and Non-drug (GAP-43 overall *p* = 0.002; S-100 overall *p* = 0.001).

Histopathological findings among groups in the acute phase are presented in Table 3. There were statistically significant differences between groups in terms of axonal continuity, Schwann cell density, myelin sheath quality, and GAP-43 and S-100 staining (*p* < 0.001) (Figure 4). However, no significant differences were observed between groups in terms of inflammatory cell and capillary structure density.

The pathological findings for the chronic phase are shown in Table 4. Statistically significant differences were detected between groups in Schwann cell density, myelin sheath quality, GAP-43, and S-100 staining (Figure 4), while no differences were noted for axonal continuity, inflammatory cell density, or capillary structure density. No significant difference was found when GAP-43 staining rates were compared between acute and chronic drug groups. However, significant differences were identified in S-100 staining rates (*p* = 0.027) (Supplementary Table S5).

## 4. Discussion

LEV is a neuromodulator that has been increasingly used as an antiepileptic agent in recent years, with several studies demonstrating its anti-inflammatory properties and its role in enhancing neuronal recovery [17,18,19]. In an experimental study by Ljiljana Djekic and colleagues, intraperitoneal administration of LEV in rats showed anti-inflammatory effects comparable to those of topically applied ibuprofen [17]. Furthermore, Huichao Zou and colleagues found that administering LEV at a dose of 50 mg/kg for 20 days had significant histological, molecular, and physical effects in promoting neuronal recovery following traumatic brain injury [18].

In our study, we administered intraperitoneal LEV at a dose of 50 mg/kg for 7 days (acute phase) and 28 days (chronic phase). To our knowledge, this is the first study to evaluate the use of LEV specifically in PN crush injury. While there was no statistically significant difference between the non-drug and drug groups in terms of SFI values and gastrocnemius muscle weight ratios during the acute period, a significant improvement in SFI values was observed in the chronic period, starting after the 14th day, between the non-drug and drug groups.

These findings suggest that while a 7-day LEV treatment may not be sufficient to promote functional recovery in the early stages of neuronal damage post-crush injury, its effects become apparent in the chronic phase. This is consistent with the findings of Feng Xinhong et al., who investigated the impact of dexamethasone on sciatic nerve injury. In their study, no improvement was observed in the SFI tests during the acute period; however, a significant improvement was noted between the drug and control groups by the 28th day [1].

In the acute phase of our study, axonal continuity was significantly greater in the drug-administered group compared to the non-drug group, suggesting that LEV has a positive effect on PN regeneration. Although this difference was not statistically significant in the chronic phase, the number of rats with complete axonal continuity increased in both the non-drug and drug groups. Schwann cell density, an indicator of regeneration, increased in the injured groups; however, while there was no statistically significant difference between the drug and non-drug groups in the acute phase, a significant increase favoring the drug group was observed in the chronic phase.

Regarding the myelin sheath, a higher level of homogeneity indicates a closer similarity to normal conditions. In the drug group, a significant improvement in myelin sheath homogeneity was observed during the acute phase. Although this improvement persisted in the chronic phase, it was not statistically significant, which may suggest that the body’s intrinsic auto-regeneration ability contributes to recovery over time.

The absence of inflammatory cell density was detected in 12.5% of the cases in the drug group during the acute phase. In the chronic phase, it was absent in 75% of the cases in the drug group, which was higher than both the control and non-drug groups. These findings indicate that LEV exhibits an anti-inflammatory effect, particularly in the chronic phase, which also contributes to nerve regeneration.

In recent years, the use of neural structure-specific proteins such as GAP-43 and S-100 has become more prominent in neuronal histopathological examinations, replacing standard staining techniques. In our study, no GAP-43 reactivity was observed in the control group, while a slight increase was seen in the non-drug group, suggesting some self-regenerative ability. In the drug group, GAP-43 reactivity was remarkably higher than in the other groups, indicating that LEV has a positive effect on nerve regeneration. This trend persisted in the chronic phase, where GAP-43 reactivity reached its highest levels.

Our findings support those of Feng Xinhong’s study on dexamethasone, which demonstrated that elevated GAP-43 levels correlated with improved nerve regeneration, though their assessment was performed after 28 days [1]. In contrast, our study showed positive outcomes in both the acute and chronic phases. Similarly, Mohammad et al.’s research on diabetic rats found that LEV increased GAP-43 expression, indicating its neuroprotective effect [7].

Accumulation of S-100 protein marks the proliferation of Schwann cells in the sciatic nerve. Proliferative Schwann cells support continuous regeneration and functional recovery of the sciatic nerves [20]. In their study on curcumin following sciatic nerve amputation, Yun-Gang Luo and colleagues observed high S-100 levels and concluded that curcumin enhances nerve regeneration [20]. Similarly, in our study, S-100 protein reactivity was higher in the drug groups during both the acute and chronic phases, suggesting that LEV promotes PN recovery.

Mechanistically, our observations can be integrated into current knowledge of peripheral nerve regeneration biology. Nerve regeneration involves axonal sprouting, remyelination, and reinnervation of target tissues, processes that are regulated by growth factors, Schwann cell activity, and axon–glia interactions. Levetiracetam binds to synaptic vesicle protein 2A (SV2A) and modulates calcium-dependent neurotransmitter release. Through these actions, it may indirectly reduce excitotoxicity and promote an environment favorable to axonal repair. In addition, levetiracetam has been shown to influence intracellular calcium dynamics, oxidative stress, and inflammatory pathways, all of which play crucial roles in peripheral nerve regeneration. The increased GAP-43 and S-100 expression observed in our study aligns with these mechanisms, suggesting that levetiracetam enhances axonal outgrowth and Schwann cell activity during the regenerative process.

Another important translational aspect is the potential role of non-invasive imaging modalities. While our study relied on histological and functional endpoints, emerging technologies such as ultra-high-resolution ultrasound now allow dynamic monitoring of peripheral nerve regeneration in vivo. Recent work by Snoj et al. demonstrated that high-resolution ultrasound can differentiate sciatic nerve fascicles with histological correlation, highlighting its potential to bridge animal model findings with human clinical applications [21]. Incorporating such imaging approaches into future studies may facilitate real-time evaluation of nerve repair and strengthen translational relevance.

In addition to the histological and functional effects observed, our findings may be linked to molecular mechanisms involving synaptic remodeling. Levetiracetam has been reported to attenuate the expression of genes regulating synaptic plasticity, including ARC (activity-regulated cytoskeleton-associated protein), which plays a crucial role in activity-dependent structural remodeling [22]. Recent studies further emphasize the importance of ARC in neural repair and functional recovery [23]. Although ARC was not assessed in our study, it is conceivable that levetiracetam’s beneficial effects on peripheral nerve regeneration may, in part, involve modulation of ARC expression.

This study has several limitations. First, although the SNI model is widely used for evaluating PN regeneration, it may not fully replicate the complexity of nerve injuries in humans. We employed a single sciatic nerve crush injury model; therefore, the applicability of our findings to other types of PN injuries (e.g., transection, stretch, or chronic compression) remains to be determined. Second, our study tested only one levetiracetam dose, and thus, dose–response relationships could not be established. Third, our functional assessments were limited to a 28-day period, and longer-term outcomes such as full reinnervation or recovery of fine motor control were not evaluated. Additionally, the assessment of nerve recovery relied on histopathological and functional tests specific to the sciatic nerve, which may not be universally applicable to other PNs. Furthermore, we did not investigate the molecular pathways underlying the observed effects of LEV, which could provide a deeper understanding of its mechanism of action. Finally, while the use of animal models offers valuable insights, translating these findings to clinical practice in humans requires further investigation through larger, controlled clinical studies.

In conclusion, this study demonstrates that LEV, which is used as an antiepileptic agent in practice, has promising effects on PN, particularly in the chronic phase following sciatic nerve injury. Administration of LEV over a longer duration showed marked improvements in axonal continuity, Schwann cell density, and myelin sheath quality. These findings suggest that LEV’s neuroprotective and anti-inflammatory properties contribute positively to the regeneration process, highlighting its potential as a therapeutic agent for PN injuries. However, further studies are necessary to explore the underlying molecular mechanisms and validate these effects in clinical settings.

## Figures and Tables

**Table 1:** Nerve regeneration evaluation scale.

Criteria	Grades
Axonal continuity on both sides of the repair area	Poor, unorganized axonal proliferation
	Moderate
	Complete continuity in the healing area
Schwann cell density	Poor
	Moderate
	Dense
Myelin Sheath	Absent or poor
	Vacuolar and poor
	Circular and homogeneous
Number of inflammatory cells	≥10
	1–9
	None
Capillary structure	≥6
	3–6
	1–3
Total	

**Table 2:** Comparison of gastrocnemius muscle ratios and Sciatic Functional Index (SFI) measurements in the acute and chronic phases.

	Groups									
	Acute Control			Acute Non-Drug			Acute Drug			*p*-Value
	Mean (X)	Standard Deviation (SD)	Median	Mean (X)	Standard Deviation (SD)	Median	Mean (X)	Standard Deviation (SD)	Median	
Right GCN	1593.1	234.4	1555.5	1302.6	172.8	1250.5	1341.8	253.8	1420.0	**0.044**
Left GCN	1575.4	261.9	1533.5	1645.1	184.4	1585.5	1717.2	196.8	1743.0	0.210
Right/Left Ratio	1.01	0.04	1.01	0.8	0.04	0.8	0.8	0.1	0.8	**<0.001**
SFI Day 7	−10.2	1.9	−10.7	−74.1	6.2	−71.9	−73.4	6.8	−71.3	**<0.001**
	**Chronic Control**			**Chronic Non-Drug**			**Chronic Drug**			
	**Mean (X)**	**Standard Deviation (SD)**	**Median**	**Mean (X)**	**Standard Deviation (SD)**	**Median**	**Mean (X)**	**Standard Deviation (SD)**	**Median**	***p*-Value**
Right GCN	2110.6	117.9	2086.0	1530.0	192.6	1488.0	1415.8	109.2	1394.0	**<0.001**
Left GCN	2069.4	221.5	2130.0	2176.2	231.9	2103.0	2068.0	189.9	2041.0	0.524
Right/Left Ratio	1.02	0.1	1.01	0.7	0.04	0.7	0.7	0.05	0.7	**<0.001**
SFI Day 7	−10.3	1.6	−10.3	−75.5	9.0	−78.7	−80.4	5.2	−80.9	**<0.001**
SFI Day 14	−8.2	2.4	−9.4	−77.4	3.8	−78.7	−70.6	8.1	−74.1	**<0.001**
SFI Day 21	−8.4	1.4	−8.8	−69.4	6.0	−69.9	−51.9	6.6	−48.8	**<0.001**
SFI Day 28	−6.9	2.2	−7.8	−49.9	46.0	−65.6	−44.0	7.7	−43.0	**0.009**

**Table 3:** Comparisons of histopathologic parameters among groups in the acute phase.

			Groups			*p*-Value
			Acute Control	Acute Non-Drug	Acute Drug	
Axonal continuity	Partial	n	0 ^a^	8 ^b^	4 ^c^	
		%	0.0	100.0	50.0	**<0.001**
	Complete	n	8 ^a^	0 ^b^	4 ^c^	
		%	100.0	0.0	50.0	
Schwann Cell Density	Moderate	n	8 ^a^	4 ^b^	3 ^b^	
		%	100.0	50.0	37.5	**0.024**
	High	n	0 ^a^	4 ^b^	5 ^b^	
		%	0.0	50.0	62.5	
Myelin Sheath	Absent or Weak	n	0 ^a^	8 ^b^	0 ^a^	
		%	0.0	100.0	0.0	
	Vacuolated and Weak	n	0 ^a^	0 ^a^	4 ^b^	**<0.001**
		%	0.0	0.0	50.0	
	Circular and Homogenous	n	8 ^a^	0 ^b^	4 ^c^	
		%	100.0	0.0	50.0	
Inflammatory Cell Density	Absent	n	0 ^a^	0 ^a^	1 ^a^	
		%	0.0	0.0	12.5	
	1–9	n	8 ^a^	5 ^a^	7 ^a^	0.083
		%	100.0	62.5	87.5	
	>10	n	0 ^a^	3 ^a^	0 ^a^	
		%	0.0	37.5	0.0	
Capillary Structure	1–3	n	1 ^a^	0 ^a^	2 ^a^	
		%	12.5	0.0	25.0	
	3–6	n	6 ^a^	4 ^a^	5 ^a^	0.293
		%	75.0	50.0	62.5	
	>6	n	1 ^a^	4 ^a^	1 ^a^	
		%	12.5	50.0	12.5	
Gap-43 Staining	0	n	8 ^a^	0 ^b^	0 ^b^	
		%	100.0	0.0	0.0	
	1–25%	n	0 ^a^	8 ^b^	1 ^a^	
		%	0.0	100.0	12.5	**<0.001**
	26–50%	n	0 ^a^	0 ^a^	4 ^b^	
		%	0.0	0.0	50.0	
	51–75%	n	0 ^a^	0 ^a^	3 ^a^	
		%	0.0	0.0	37.5	
S-100 Staining	1	n	8 ^a^	0 ^b^	1 ^b^	
		%	100.0	0.0	12.5	
	2	n	0 ^a^	1 ^a^	0 ^a^	**<0.001**
		%	0.0	12.5	0.0	
	3	n	0 ^a^	4 ^b^	3 ^a, b^	
		%	0.0	50.0	37.5	
	4	n	0 ^a^	3 ^a, b^	4 ^b^	
		%	0.0	37.5	50.0	

^a^, ^b^, ^c^ symbols indicate cells where there is a statistical difference. Cells with different symbols show a statistically significant difference between them.

**Table 4:** Comparisons of histopathologic parameters among groups in the chronic phase.

			Groups			*p*-Value
			Chronic Control	Chronic Non-drug	Chronic Drug	
Axonal continuity	Partial	n	0 ^a^	3 ^a^	0 ^a^	
		%	0.0	37.5	0.0	0.083
	Complete	n	8 ^a^	5 ^a^	8 ^a^	
		%	100.0	62.5	100.0	
Schwann Cell Density	Moderate	n	8 ^a^	4 ^b^	0 ^c^	
		%	100.0	50.0	0.0	**<0.001**
	High	n	0 ^a^	4 ^b^	8 ^c^	
		%	0.0	50.0	100.0	
Myelin Sheath	Absent or Weak	n	0 ^a^	1 ^a^	0 ^a^	
		%	0.0	12.5	0.0	
	Vacuolated and Weak	n	0 ^a^	5 ^b^	8 ^b^	**<0.001**
		%	0.0	62.5	100.0	
	Circular and Homogenous	n	8 ^a^	2 ^b^	0 ^b^	
		%	100.0	25.0	0.0	
Inflammatory Cell Density	Yok	n	5 ^a^	3 ^a^	6 ^a^	
		%	62.5	37.5	75.0	
	1–9	n	3 ^a^	3 ^a^	2 ^a^	0.368
		%	37.5	37.5	25.0	
	>10	n	0 ^a^	2 ^a^	0 ^a^	
		%	0.0	25.0	0.0	
Capillary Structure	1–3	n	1 ^a^	0 ^a^	0 ^a^	
		%	12.5	0.0	0.0	
	3–6	n	7 ^a^	6 ^a^	8 ^a^	0.304
		%	87.5	75.0	100.0	
	>6	n	0 ^a^	2 ^a^	0 ^a^	
		%	0.0	25.0	0.0	
Gap43 Staining	0	n	6 ^a^	2 ^b^	0 ^b^	
		%	75.0	25.0	0.0	
	1–25%	n	2 ^a^	6 ^b^	4 ^a, b^	
		%	25.0	75.0	50.0	**0.002**
	26–50%	n	0 ^a^	0 ^a^	3 ^a^	
		%	0.0	0.0	37.5	
	76–100%	n	0 ^a^	0 ^a^	1 ^a^	
		%	0.0	0.0	12.5	
S100 Staining	1	n	8 ^a^	3 ^b^	1 ^b^	
		%	100.0	37.5	12.5	
	2	n	0 ^a^	5 ^b^	5 ^b^	**0.001**
		%	0.0	62.5	62.5	
	3	n	0 ^a^	0 ^a^	2 ^a^	
		%	0.0	0.0	25.0	

^a^, ^b^, ^c^ symbols indicate cells where there is a statistical difference. Cells with different symbols show a statistically significant difference between them.

## Data Availability

The datasets used and/or analyzed during the current study available from the corresponding author on reasonable request.

## Supplementary Materials

The following supporting information can be downloaded at: https://www.mdpi.com/article/10.3390/jcm14186374/s1, Supplementary Table S1. Post hoc analysis of gastrocnemius muscle ratios and Sciatic Functional Index (SFI) measurements in the acute group; Supplementary Table S2. Post hoc analysis of gastrocnemius muscle ratios and Sciatic Functional Index (SFI) measurements in the chronic group; Supplementary Table S3. Comparison of SFI values between groups in the chronic phase by days; Supplementary Table S4. Comparison of SFI and muscle ratios between groups in the acute and chronic phases; Supplementary Table S5. Comparison of GAP43 Staining Between Acute and Chronic Drug Groups.
