# Supplementary material for: Evaluating the Neuroprotective Effects of Levetiracetam on Experimental Sciatic Nerve Injury
Source: J Clin Med. 2025 Sep 10;14(18):6374. doi: 10.3390/jcm14186374 (PMC12470501; doi:10.3390/jcm14186374)
Supplement: Supplementary file 1 [file jcm-14-06374-s001.zip › jcm-3830415-supplementary.pdf]

Supplementary Table 1. Post-hoc analysis of gastrocnemius muscle ratios and Sciatic Functional Index (SFI) measurements in the acute group

Variable	(I) GRUP NO	(J) GRUP NO	Mean Difference (I-J)	Std. Error	p-value
Right/Left Ratio	Acute Control	Acute Sham	0,222805*	0,037853	<0,001
		Acute Drug	0,232910*	0,037853	<0,001
	Acute Sham	Acute Control	-0,222805*	0,037853	<0,001
		Acute Drug	0,010105	0,037853	1,000
	Acute Drug	Acute Control	-0,232910*	0,037853	<0,001
		Acute Sham	-0,010105	0,037853	1,000
SFI Day 7	Acute Control	Acute Sham	63,81125*	2,70788	<0,001
		Acute Drug	63,18750*	2,70788	<0,001
	Acute Sham	Acute Control	-63,81125*	2,70788	<0,001
		Acute Drug	-0,62375	2,70788	1,000
	Acute Drug	Acute Control	-63,18750*	2,70788	<0,001
		Acute Sham	0,62375	2,70788	1,000

* Indicate cells where there is a statistical difference.

Supplementary Table 2. Post-hoc analysis of gastrocnemius muscle ratios and Sciatic Functional Index (SFI) measurements in the chronic group

Variable	(I) GRUP NO	(J) GRUP NO	Mean Difference (I-J)	Std. Error	p-value
Right/Left Ratio	Chronic Control	Chronic Sham	,325*	,036197	<0,001
		Chronic Drug	,342*	,036197	<0,001
	Chronic Sham	Chronic Control	-,325*	,036197	<0,001
		Chronic Drug	,0163	,036197	1,000
	Chronic Drug	Chronic Control	-,342057*	,036197	<0,001
		Chronic Sham	-,016336	,036197	1,000
SFI Day 7	Chronic Control	Chronic Sham	65,18750*	3,04409	<0,001
		Chronic Drug	70,11375*	3,04409	<0,001
	Chronic Sham	Chronic Control	-65,18750*	3,04409	<0,001
		Chronic Drug	4,92625	3,04409	0,362
	Chronic Drug	Chronic Control	-70,11375*	3,04409	<0,001
		Chronic Sham	-4,92625	3,04409	0,362
SFI Day 14	Chronic Control	Chronic Sham	69,19000*	2,68986	<0,001
		Chronic Drug	62,41125*	2,68986	<0,001
	Chronic Sham	Chronic Control	-69,19000*	2,68986	<0,001
		Chronic Drug	-6,77875	2,68986	0,060
	Chronic Drug	Chronic Control	-62,41125*	2,68986	<0,001
		Chronic Sham	6,77875	2,68986	0,060
SFI Day 21	Chronic Control	Chronic Sham	61,00875*	2,60308	<0,001
		Chronic Drug	43,46625*	2,60308	<0,001
	Chronic Sham	Chronic Control	-61,00875*	2,60308	<0,001
		Chronic Drug	-17,54250*	2,60308	<0,001
	Chronic Drug	Chronic Control	-43,46625*	2,60308	<0,001
		Chronic Sham	17,54250*	2,60308	<0,001
SFI Day 28	Chronic Control	Chronic Sham	43,04625*	13,48312	0,013
		Chronic Drug	37,11625*	13,48312	0,036
	Chronic Sham	Chronic Control	-43,04625*	13,48312	0,013
		Chronic Drug	-5,93000	13,48312	1,000
	Chronic Drug	Chronic Control	-37,11625*	13,48312	0,036
		Chronic Sham	5,93000	13,48312	1,000

* Indicate cells where there is a statistical difference.

Supplementary Table 3. Comparison of SFI values between groups in the chronic phase by days

	SFI Day 7	SFI Day 14	SFI Day 21	SFI Day 28	p-value
**Chronic Control**					
Mean (X)	-10,3350	-8,1900	-8,4063	-6,9000	
Standard Deviation (SD)	1,55606	2,36702	1,41523	2,25915	**0.019**
Median	-10,2850	-9,3950	-8,8450	-7,7800	
**Chronic Sham**					
Mean (X)	-75,5225	-77,3800	-69,4150	-49,9462	
Standard Deviation (SD)	9,01378	3,79166	6,01472	46,01883	**0.006**
Median	-78,6800	-78,6800	-69,9000	-65,6200	
**Chronic Drug**					
Mean (X)	-80,4488	-70,6013	-51,8725	-44,0163	
Standard Deviation (SD)	5,24671	8,17586	6,56755	7,66143	**<0.001**
Median	-80,9450	-74,0650	-48,8550	-43,0250	

Supplementary Table 4. Comparison of SFI and muscle ratios between groups in the acute and chronic phases

	GROUPS						
	Acute Control			Chronic Control			
	Mean (X)	Standard Deviation (SD)	Median	Mean (X)	Standard Deviation (SD)	Median	p-value
SFI Day 7	-10,2463	1,946	-10,72	-10,33	1,55	-10,28	0.921
Right/Left Ratio	1,01408	0,038	1,01	1,02	0,107	1,00	0.721
	Acute Sham			Chronic Sham			
SFI Day 7	-74,05	6,21	-71,90	-75,52	9,01	-78,68	0.711
Right/Left Ratio	0,79	0,043	0,788	0,703	0,045	0,705	**0.001**
	Acute Drug			Chronic Drug			
SFI Day 7	-73,43	6,75	-71,33	-80,44	5,24	-80,94	0.036
Right/Left Ratio	0,78	0,117	0,79	0,68	0,046	0,67	0.053

Supplementary Table 5. Comparison of GAP43 Staining Between Acute and Chronic Drug Groups

			**GROUPS**		
			**Acute Drug**	**Chronic Drug**	**p-value**
GAP43 Staining	%0-25	n	1_a	4_a	
		%	12,5%	50,0%	
	%26-50	n	4_a	3_a	
		%	50,0%	37,5%	0.114
	%51-75	n	3_a	0_a	
		%	37,5%	0,0%	
	%76-100	n	0_a	1_a	
		%	0,0%	12,5%	
S100 Staining	1	n	1_a	1_a	
		%	12,5	12,5	
	2	n	0_a	5_b	**0.027**
		%	0,0	62,5	
	3	n	3_a	2_a	
		%	37,5	25,0	
	4	n	4_a	0_b	
		%	50,0	0,0	

*, a, b, c symbols indicate cells where there is a statistical difference. Cells with different symbols show a statistically significant difference between them.
